# Chronic intermittent hypoxia-induced hypertension: the impact of sex hormones

**DOI:** 10.1152/ajpregu.00258.2023

**Published:** 2024-02-26

**Authors:** Cephas B. Appiah, Jennifer J. Gardner, George E. Farmer, Rebecca L. Cunningham, J. Thomas Cunningham

**Affiliations:** ^1^Department of Physiology and Anatomy, School of Biomedical Sciences, University of North Texas Health Science Center at Fort Worth, Fort Worth, Texas, United States; ^2^Department of Pharmaceutical Sciences, System College of Pharmacy, University of North Texas Health Science Center at Fort Worth, Fort Worth, Texas, United States

**Keywords:** estrogens, hypertension, sleep apnea, sympathetic nervous system, testosterone

## Abstract

Obstructive sleep apnea, a common form of sleep-disordered breathing, is characterized by intermittent cessations of breathing that reduce blood oxygen levels and contribute to the development of hypertension. Hypertension is a major complication of obstructive sleep apnea that elevates the risk of end-organ damage. Premenopausal women have a lower prevalence of obstructive sleep apnea and cardiovascular disease than men and postmenopausal women, suggesting that sex hormones play a role in the pathophysiology of sleep apnea-related hypertension. The lack of protection in men and postmenopausal women implicates estrogen and progesterone as protective agents but testosterone as a permissive agent in sleep apnea-induced hypertension. A better understanding of how sex hormones contribute to the pathophysiology of sleep apnea-induced hypertension is important for future research and possible hormone-based interventions. The effect of sex on the pathophysiology of sleep apnea and associated intermittent hypoxia-induced hypertension is of important consideration in the screening, diagnosis, and treatment of the disease and its cardiovascular complications. This review summarizes our current understanding of the impact of sex hormones on blood pressure regulation in sleep apnea with a focus on sex differences.

## INTRODUCTION

Obstructive sleep apnea (OSA) is a sleep-related disorder characterized by recurrent upper airway collapse, resulting in episodic reductions in breathing. OSA can be measured by the apnea/hypopnea index (AHI), the total number of apneic or hypopneic episodes per hour during sleep with cut-off points of 5–15, 16–30, and >30 corresponding to mild, moderate, and severe forms, respectively (1). OSA is an important risk factor for cardiovascular and metabolic disorders and severely impacts the quality of life of affected individuals due to apnea/hypopnea, sleep fragmentation, snoring, microarousals, and insomnia, resulting in daytime somnolence and lethargy (2–5).

Globally, it is estimated that ∼936 million adults between 39 and 69 yr old suffer from OSA, of which 425 million are affected by moderate to severe forms (6). The prevalence of OSA in men and women 30–70 yr of age in the United States increased by 14% to 55% from 1993 to 2013 (7). Between the ages of 30 and 49 yr, premenopausal women show an estimated OSA prevalence of 3%, whereas men in the same age range have a prevalence of 10%, indicating a more than threefold increase in men as compared with premenopausal women (7). In older men aged 50 to 70 yr, the estimated prevalence is ∼17% representing about a twofold increase as compared with younger men (30–49 yr) (7). In postmenopausal women in the same age bracket (50–70 yr), the estimated prevalence of OSA is 9%, representing a threefold increase as compared with premenopausal women 30–49 yr of age (7). Thus, independent of sex, the prevalence of OSA increases with age, but women experience a greater increase (threefold) than men (twofold), which narrows the difference in the prevalence between men and postmenopausal women of the same age (7, 8). This suggests that sex hormonal changes associated with aging may play a role in sleep apnea and related complications. Although OSA prevalence substantially increases in postmenopausal women, the increase does not align with age-matched males. There is currently a lack of longitudinal studies tracking both sexes to determine how age-related depletion of sex hormones or genetic sex differences influence OSA onset with aging. In addition, underreporting and underdiagnosis likely contribute to an underestimate of OSA incidence in older females (9–11).

OSA exhibits a complex disease phenotype with multifactorial mechanisms of pathology encompassing intermittent hypoxia, hypercapnia, inflammation, and deleterious changes in metabolic function that lead to cardiovascular, neurological, and metabolic impairments (4, 12–14). Patients with OSA have an increased risk of developing treatment-resistant hypertension, diabetes, altered lipid metabolism, and other metabolic dysfunctions (4, 5, 15, 16). Although OSA significantly impacts both men and women, sex-based differences regarding the nature of apnea/hypopnea episodes, alterations in sex hormone concentrations, and level of daytime somnolence have been identified (10, 17–22). In patients with moderate to severe OSA, men have greater oxygen desaturation and longer apnea and hypopnea events compared with women with a similar AHI (10, 17–19, 22). Thus, the AHI has limited accuracy in classifying the actual severity of OSA, especially in comparisons between sexes. This important limitation needs to be considered in OSA outcomes in sex-based studies. The higher prevalence of OSA in men and postmenopausal women compared with premenopausal women suggests that estrogen and progesterone insufficiency may account for the increased susceptibility to the disease (10, 23–25). Even with a similar cardiovascular risk, men show a higher sympathovagal balance, indicative of autonomic dysfunction, relative to postmenopausal women with matched OSA severity, highlighting sex differences in the disease outcomes (26). Studies regarding OSA hypertension imply a relationship between OSA hypertension and sex (27). Yet factors such as delayed diagnosis, underdiagnosis, comorbidities, variability in oxygen desaturation, and clinical subtype heterogeneity in men and women remain important limitations associated with many studies (2). Even with these challenges, additional insights into the pathophysiological mechanisms and sex-based differences associated with OSA hypertension have been gained through animal models using chronic intermittent hypoxia (CIH) paradigms that simulate the repetitive cycles of hypoxia in OSA.

Sex hormones may influence the central nervous system (CNS) and peripheral systems that promote CIH hypertension. Central autonomic control regions in the hypothalamus and hindbrain, as well as peripheral mechanisms such as the renin-angiotensin system (RAS), are involved in cardiovascular homeostasis (28, 29). Testosterone appears to facilitate the hypertensive response in intermittent hypoxia through the upregulation of RAS activity, the amplification of renal and vascular responses via signaling pathways mediated by Rho kinase, *c-Src*, NADPH-oxidase (NOX), and the membrane androgen receptor (30–33). Estrogen and progesterone, on the other hand, confer cardiovascular protection (34–36). Elucidating the molecular pathways involved has important implications for understanding sex disparities in OSA outcomes and developing appropriate screening and management strategies. This review summarizes current evidence on the impact of sex hormones in intermittent hypoxia-induced hypertension, with a focus on insights from preclinical studies.

## CHRONIC INTERMITTENT HYPOXIA MODEL AND OBSTRUCTIVE SLEEP APNEA

CIH can be used to simulate the hypoxia that occurs in OSA (37). First introduced by Fletcher and colleagues, CIH is a widely employed rodent model that generates intermittent hypoxia-induced hypertension dependent on the intensity of the CIH exposure (37–40). The wide variation in CIH protocols in animal research facilitates a deeper understanding of the impact of intermittent hypoxia intensity on physiological function and reflects the natural extensive spectrum of apnea/hypopnea event intensity and frequency in patients with OSA. Of note, strain and housing conditions can affect CIH outcomes such as weight, oxidative stress, and circulating hormones (41). The pathogenicity of CIH is primarily dependent on the degree of hypoxia and the number of cycles of intermittent hypoxia applied (37).

Independent of hypercapnia, CIH causes sympathetic nerve hyperactivity, increased cardiac output, vasoconstriction, and enhanced activation of the RAS, leading to sustained elevation in mean arterial pressure (MAP) in male rats (28, 29, 39, 40, 42). A single episode of intermittent hypoxia comprised 45 s of hypoxia (8% $\text{F}{\text{I}}_{{\text{O}}_{2}}$) cycled with 5 min of normoxia (21% $\text{F}{\text{I}}_{{\text{O}}_{2}}$) increases sympathetic nerve discharge and phrenic nerve activity acutely in male rats (43). Nine repeated episodes following the initial stimulation induced long-term facilitation in the sympathetic and respiratory motor nerve activity after the cessation of the stimulation and normalization of blood gases (43).

CIH alters peripheral chemoreceptor sensitivity, which contributes to exaggerated sympathetic activation (40, 42, 44, 45). Carotid body denervation abrogates CIH hypertension highlighting the importance of carotid afferent signaling to CIH hypertension (44, 46, 47). CIH increases the activation of the central autonomic regulatory regions in male Sprague-Dawley rats supporting the increase in sympathetic activation and MAP (28). Furthermore, CIH alters vascular reactivity, impairs endothelium-dependent vasorelaxation, and promotes vascular remodeling in males (48). Seven days of CIH treatment increases FosB staining in the hypothalamic lamina terminalis and hindbrain preautonomic regions and upregulates the expression of angiotensin II type 1 receptor (AT1R) and nitric oxide synthase 1 (NOS1) to increase excitatory neuronal signaling that facilitates sustained increase in MAP in male rats (28, 49–51). Reoxygenation from CIH generates proinflammatory reactive oxygen species (ROS) triggering inflammation and oxidative stress, which may contribute to CNS changes and vascular dysfunction that support CIH hypertension in males (52–54).

In summary, CIH increases activation of central autonomic regions that regulate MAP, generates ROS, promotes cardiovascular dysfunction, alters nitric oxide bioavailability, and exaggerates sympathetic activity resulting in CIH hypertension. However, most of the early studies used only male rats (38–40, 46). Although this allows characterization of pathways in a susceptible population, the lack of equal integration of parallel female cohorts limits the interpretability and translatability of the findings. Purposeful and methodical testing of sex as a biological variable in CIH studies will advance the identification of novel areas of biological divergence. Such studies will illuminate varied therapeutic targets or preventative strategies between males and females.

## SEX HORMONES, OBSTRUCTIVE SLEEP APNEA, AND CARDIOVASCULAR DISEASE

The difference in clinical symptomatology between men, premenopausal women, and postmenopausal women may contribute to sex-based differences in the diagnosis of OSA (10, 11). Men present with OSA symptoms related to cardiovascular and metabolic function like diurnal hypertension, snoring, and daytime somnolence, whereas women generally experience nonspecific symptoms such as mood disturbances, morning headaches, fatigue with a reduction in work performance, and poor quality of life (11, 23, 55–58). Nonetheless, lower serum concentration of estrogen and progesterone in postmenopausal women is associated with snoring and choking, which are typical symptoms in males with OSA (24, 59). These differences in presentation of the disease may promote misdiagnosis or underdiagnosis in premenopausal women. Premenopausal females have a lower prevalence of OSA than in men and postmenopausal women, suggesting that sex hormones may be important to the pathophysiology of OSA (24, 25). With the abrupt decline in estrogen but preserved ovarian and adrenal androgen production, postmenopausal women develop a hormonal shift from a premenopausal estrogenic state to a predominant postmenopausal androgenic state (60). Thus, the increased predisposition to sleep apnea, systemic oxidative stress, and neurological and cardiovascular dysfunction may not only stem from a precipitous drop in estrogen but also the shift in the premenopausal sex-steroid profile (60, 61).

OSA is associated with impaired testosterone secretion and circadian rhythms in men, with greater hypogonadal effects observed in severe OSA cases (62). However, in premenopausal women, decrements in estrogen and progesterone assessed during early follicular phase have only been evidenced in patients with severe OSA, not milder forms (63). In postmenopausal patients already exhibiting low sex steroid levels, a further reduction of progesterone but not estrogen was associated with OSA (63). It should be noted, however, that assessing hormonal status exclusively in the early follicular phase likely fails to capture peak levels occurring later in the cycle.

### Studies on the Impact of Sex Hormone Depletion and Replacement Therapies in Men and Women

Premature reduction in ovarian hormones induced by bilateral oophorectomy has been associated with multiple adverse health outcomes in premenopausal women, likely mediated by the abrupt depletion of sex hormones (64–66). Studies have shown an increased risk of OSA, dyslipidemia, cardiovascular disease morbidity, and mortality in oophorectomized patients compared with premenopausal controls with intact ovarian function (64–66). The initiation of postoperative hormone replacement therapy (HRT) ideally within 1 yr after surgical menopause, continuing until median age of natural menopause (∼50 yr of age), appears to mitigate a significant portion of the long-term cardiovascular and metabolic morbidity associated with oophorectomy (64–66). In postmenopausal women, hormone replacement therapy with either estradiol or a combination of estradiol and medroxyprogesterone acetate reduces the respiratory distress index, the number of waking episodes, and improves sleep quality, total sleep time, resting ventilation, and hypoxic ventilatory response (HVR) (67–72). These respiratory optimizing effects of HRT may attenuate the impact of OSA and subsequent hypertension in postmenopausal women.

In contrast, clinical data assessing impacts of androgen deprivation therapy (ADT), through either bilateral orchiectomy or pharmacological testosterone suppression, has revealed inconsistent cardiovascular effects in treated men (73). Although some studies demonstrate an inverse relationship between circulating testosterone levels and blood pressure in ADT groups, others report an overall cardiovascular benefit from induced hypogonadism (73–75). Potential explanations for these discordant outcomes likely include differences in the patient age distributions, degree of baseline testosterone deficiency before ADT initiation, and an absence of properly matched control groups (73). Additional longitudinal studies controlling for critical demographic variables and endogenous hormonal confounders at baseline are necessary to clarify the complex dose-responsive effects of androgen deprivation on cardiovascular health over time. Furthermore, the adoption of standardized ADT treatment guidelines tailored to patient endocrine profiles may help reconcile these ambiguous findings in clinical literature.

There are limited clinical data elucidating the relationship between testosterone levels and OSA. Low circulating testosterone and testosterone replacement therapy (TRT) have been reported to have variable effects on OSA possibly due to differences in the extent of testosterone deficiency or dose of exogenous testosterone administered in TRT (76–78). Physiologically titrated testosterone replacement protocols based on the patient’s pretreatment endocrine profile are needed to accurately assess the therapeutic impact of TRT on sleep apnea outcomes. Further research is needed to provide clarity on the role of testosterone in OSA given the current state of the literature.

### The Association between Sex and Sleep Apnea Hypertension

Many clinical studies investigating the relationship between hypertension and OSA focused on males and primarily centered on the association between OSA severity and hypertension rather than elucidating sex-based differences in OSA-induced hypertension. As such, our understanding of sex-based differences in OSA hypertension is limited. Prospective studies such as the Wisconsin Sleep Cohort Study showed that MAP increases linearly with AHI independent of age, sex, and body mass index (79–81). However, the literature related to sex-based differences and the incidence of OSA hypertension remains inconclusive. This may be due to low compliance, attrition of study participants in lengthy follow-up studies, confounding factors related to sex and gender (for example, age, body mass index, lifestyle factors—alcohol intake, smoking, physical activity, etc.), and biased representation of sex in scientific studies. Controlled prospective analyses, powered to detect sex differences that account for major demographic and lifestyle variables are needed to clarify how sex and gender may modulate OSA and its relationship with cardiovascular disease.

## SEX HORMONES AND CHRONIC INTERMITTENT HYPOXIA-INDUCED HYPERTENSION

There is evidence of sex-based differences in CIH hypertension from preclinical studies. Exposing male rats to a 7-day CIH protocol (10% O_2_ cycled with 21% O_2_, 10 episodes/h for 8 h/day) increases blood pressure during the period of application of the intermittent hypoxia stimulus and this increase in MAP is sustained throughout the rest of the normoxic diurnal cycle (28, 49, 51). Thus, this model of CIH causes a sustained increase in the MAP that outlasts the period of application of the intermittent hypoxia in males (28, 49, 51). However, Hinojosa-Laborde and Mifflin found no increase in MAP following similar 7-day CIH (10% and 21% $\text{F}{\text{I}}_{{\text{O}}_{2}}$ cycles, 10 episodes/h for 8 h/day) in freely moving intact females based on radiotelemetry measures (51). While males are noticeably hypertensive with an average increase in MAP of 5–8 mmHg, the increase in gonadally intact females is less than 2 mmHg (28, 51). After bilateral ovariectomy, CIH exposure increased MAP in females like males (51), suggesting a protective effect of ovarian hormones against CIH hypertension.

Studies involving an extreme CIH protocol (6% and 21% ${\text{F}\text{I}}_{{\text{O}}_{2}}$ cycles, 6.6 episodes/h for 8 h/day for 35 days) observed a significant increase in the MAP of intact young adult female Wistar rats (82). In contrast to the report of Hinojosa-Laborde and Mifflin (51), recent work by Ribon-Demars et al. (83) using a comparable 7-day CIH protocol (10% O_2_ cycled with 21% O_2,_ 10 episodes/h for 8 h/day) showed a significant elevation in blood pressure in intact young adult female Sprague-Dawley rats using single time point tail-cuff plethysmography. In the same study, a separate group of intact young adult female subjects was exposed to a longer 35-day protocol with the same hypoxia cycling parameters (83). This extended CIH exposure produced a similar increase in blood pressure to that observed in another group of females exposed to CIH for 7 days in the same study (83). The differences in the results between the Ribon-Demars (83) study and the Hinojosa-Laborde and Mifflin (51) study could be due to differences in housing, restraint stress, or ambulatory versus single occasion blood pressure measurement between studies. Moreover, lack of accounting for estrous cycling and related hormonal fluctuations may have obscured female protection. It is important to consider the potential influence of the estrous cycle on MAP in CIH studies. However, such research is lacking, and the findings regarding the effects of the estrous cycle on MAP in untreated intact females are mixed. Although one study reported variations in night-time MAP with estrous cycling in intact female Wistar-Imamichi rats, other studies in intact female spontaneously hypertensive rats (SHR), Wistar-Kyoto rats, and C57BL/6 mice showed that MAP did not significantly differ across the different stages of estrous cycle (84–87). Further research is required to understand the potential impact of estrous cycling on MAP during CIH exposure to address the current knowledge gap. Importantly though, in studies with ovariectomized female rats, estrogen replacement prevented hypertension in groups exposed to either 7- or 35-day CIH (83). These findings strongly support an estrogen-mediated protective effect (83). To summarize, although direct study comparisons are complicated by variations in experimental conditions and techniques, it appears that protection against CIH hypertension in intact female rats may be dependent on CIH exposure severity and ovarian hormones, especially estrogen.

### Impact of Sex Chromosomes on Hypertension

Emerging evidence suggests sex effects on hypertension pathogenesis extend beyond simply gonadal hormone impacts to also include genetic influences of sex chromosomes in a strain-dependent manner. Interestingly, Ely et al. (88, 89) reported that the sex-determining region Y (*Sry*) gene on the *Y* chromosome in SHR associates with exaggerated sympathetic tone and higher arterial pressures. Using the four-core genotype mouse model to segregate gonadal versus chromosomal contributions, Ji et al. (90) demonstrated *XX* sex chromosome complement led to greater mean arterial pressure elevation compared with *XY* after 2 wk of angiotensin II treatment, independent of gonadal status [see review by Sandberg and Ji (91)]. This suggests that in the absence of hormonal protection, postmenopausal women may exhibit heightened pressor sensitivity compared with men. There is a lack of studies leveraging the four-core genotype approach to isolate sex chromosomal versus hormonal impacts on cardiovascular outcomes in the context of CIH-induced hypertension. Investigations using this model would be valuable to delineate whether genetic sex dissimilarities at the chromosome level may be contributing to differential hypertensive responses to CIH beyond overt gonadal effects. Findings could have translational relevance given sex differences in OSA prevalence and cardiovascular morbidity.

## IMPACT OF TESTOSTERONE

### Chronic Intermittent Hypoxia Alters Testosterone Levels and Arterial Pressure in an Age-Dependent Manner

Endogenous testosterone levels tend to decline with aging in men (60). Animal studies exploring the impacts of CIH on testosterone have reported variable results dependent on age, strain, and CIH protocol used in the study. Specifically, 8 days of CIH reduced testosterone in young adult but not middle-aged male F344/BN F1 hybrid rats, with a trend toward increasing testosterone in the middle-aged male rats (92). Paradoxically, studies in young adult male Sprague-Dawley rats found increased testosterone in studies that used CIH paradigms that are not associated with OSA phenotypes (93, 94). It remains to be determined whether using CIH protocols that more closely recapitulate OSA severity impacts testosterone status in Sprague-Dawley rats in a similar manner as those outside the typical AHI range (92). The differential effects across strains, ages, and hypoxia intensities highlight a possible complex relationship between hypothalamic-pituitary-gonadal axis adaptation and CIH stimulus patterns. The lack of data exploring strain-specific and age-related hormonal responses to CIH severely limits understanding of how these variables influence circulating testosterone. More research focusing on these variables is needed to clarify these findings.

### Impact of CIH on Arterial Pressure in Males

Several CIH exposure paradigms induce hypertension across various adult male rodent strains and species (95). Studies specifically investigating the role of testosterone status in modulating arterial pressure responses to CIH are notably lacking. As such, it is difficult to quantify the precise contribution of testosterone to CIH hypertension. However, numerous reports demonstrate increased MAP in male animals following CIH. Exposing male Wistar rats to a 14-day CIH protocol with an AHI equivalent to 30 induced hypertension in the normotensive young adult rats (96). Enhanced sympathetic tone and catecholamine levels mechanistically promoted hypertension (96). Our own laboratory has also consistently shown development of hypertension in adult male Sprague-Dawley rats following even modest CIH exposures (28, 49, 50, 97, 98).

Studies investigating perinatal CIH exposure show differential effects on blood pressure in a strain-dependent manner. Exposing newborn male SHRs to CIH (10% O_2_ cycling 21% every 90 s, 12 h/day, postnatal days 4 to 30) accelerates age-related hypertension during early adulthood (99). However, a study from the same group examining the effects of perinatal CIH in young Sprague-Dawley rats did not observe hypertension in young adult males (100), highlighting strain differences in pressor responses to perinatal CIH. Although the resting blood pressure and heart rate were unaffected in the Sprague-Dawley rats, the 30-day postnatal CIH exposure caused persistent attenuation of arterial baroreflex sensitivity control of heart rate, observed even into adulthood. Postnatal CIH exposure was associated with a ∼50% reduction in the heart rate responses to increases in blood pressure produced by phenylephrine as compared with the responses of normoxic controls. CIH-induced decreases in sensitivity were not further worsened by reexposure to intermittent hypoxia in adulthood. This baroreflex impairment associated with corresponding losses of vagal nerve projections to the heart, which matures gradually over the postnatal timeframe. Prenatal IH exposure elicited less pronounced effects on baroreflex function, likely from reduced oxidative stress versus direct neonatal exposures (100). These findings indicate that neonatal intermittent hypoxia during critical periods of development can induce chronic changes in autonomic function and impair cardiovascular reflexes thereby inducing lifelong cardiovascular vulnerability.

### Impact of Testosterone on Arterial Pressure in Other Rodent Models

Outside of intermittent hypoxia studies, compelling evidence demonstrates testosterone exerts hypertensive actions in other animal models. Both castration and androgen receptor antagonism mitigate blood pressure rise in young adult male SHR, whereas 5α-reductase inhibition had no effect (101). These results indicate testosterone acts via the androgen receptor to promote the hypertensive SHR phenotype (101). Further studies showed testosterone administration induces hypertension in both gonadectomized male and female SHRs, supportive of a causal role (102, 103). Earlier findings also suggest there may be an organizational window in early development where testosterone programs long-term elevation of arterial pressure in SHR (104). Experiments involving orchiectomy or pharmacological testosterone receptor antagonism with cyproterone acetate or flutamide reduced blood pressure in the young male (9 wk of age) SHRs (104). However, unless administered within 10 days after birth, the testosterone receptor antagonists had no effect on the hypertension of older male SHRs (25 wk) (104).

Evidence suggests testosterone’s role in hypertension is dependent on factors like genetics, age, and rodent strain. Dalmasso et al. (105) showed testosterone supplementation increased blood pressure in young adult (12 wk) male SHR yet reduced blood pressure in older (21–22 mo) males. In addition, prepubertal orchiectomy prevented age-related blood pressure elevation in male SHRs, supporting a hypertensive action of endogenous testosterone in this genetic model (102). Conversely, testosterone protected against hypertension in adult male normotensive Sprague-Dawley rats, unlike effects in age-matched SHR. This indicates hypertensive sequelae may involve complex interactions between testosterone and genetic susceptibility (106). The Sprague-Dawley phenotype resembles clinical data demonstrating inverse relationships between testosterone and blood pressure (74, 75). These observed strain differences in rodents may have clinical relevance; the blood pressure benefits of testosterone in normotensive animals mirror human studies showing inverse testosterone-hypertension associations, whereas studies using SHRs lend plausibility that testosterone deficiency could mitigate early hypertension in genetically predisposed men. However, later in life, declining testosterone may elevate cardiovascular risk. Understanding how CIH influences these genetic models could be key to translating insights on susceptibility windows for testosterone’s bimodal cardiovascular actions.

### Impact of Testosterone on the Renin-Angiotensin System

Testosterone impacts angiotensin II activity, thereby influencing blood pressure regulation. Sexual dimorphism in blood pressure, plasma renin activity (PRA), and hepatic angiotensinogen mRNA expression in gonadally intact SHR were reported by Chen and colleagues (30). Orchiectomy in SHR reduces blood pressure rises by decreasing renal and hepatic angiotensinogen expression and PRA. These antihypertensive effects are reversed with testosterone replacement (30). In contrast, ovariectomized female SHRs exhibit lowered angiotensinogen but unaffected blood pressure unless exogenous testosterone is provided, which then increases PRA, angiotensinogen, and arterial pressure (30). However, testosterone elicits the opposite responses on the renin-angiotensin axis in normotensive Sprague-Dawley rats, attenuating multiple angiotensin-related indices (106). As such, depletion of endogenous testosterone by CIH could promote blood pressure elevation in this strain by removing this suppression of the renin-angiotensin system. The strain-specific effects of testosterone on renal function appear to influence blood pressure through the renin-angiotensin system based on a genetic predisposition.

### Impact of Testosterone on Vascular Function, Oxidative Stress, and Central Autonomic Activity

At the vascular level, testosterone predominantly potentiates angiotensin II-mediated vascular reactivity and pro-oxidant effects (32, 107, 108). By supporting RAS activation, testosterone contributes to oxidative stress via angiotensin II-mediated ROS production. Males show a higher basal expression of pro-oxidants but have a lower antioxidant capacity resulting in greater susceptibility to oxidative stress than females in several rodent models and human studies (109–112). Testosterone increases vascular reactivity through the overexpression of AT1R and an increase in the vascular AT1R-to-AT2R ratio in male SHR and Wistar rats (107, 108). It also influences vascular hypertrophy and increases vascular cell ROS production, which primes the stage for development and subsequent aggravation of arterial hypertension in male SHR and Wistar rats (31). Testosterone-induced oxidative stress is mediated via the membrane androgen receptor – NOX complex and AT1R-dependent NOX generation of ROS (33, 108).

Interestingly, other studies have reported protective effects of testosterone on oxidative stress and vascular function, suggesting that testosterone exhibits binary effects on the vasculature depending on age, strain, basal oxidative stress, and NOS activity (113, 114). Testosterone suppresses mitochondrial dysfunction and protects cardiomyocytes against oxidative stress (115–117). Notwithstanding, the antioxidant activity of testosterone appears to be thwarted in an environment with high basal oxidative stress levels beyond the specific threshold of testosterone’s antioxidant capacity (60, 118, 119). In addition, low physiological testosterone levels can exacerbate oxidative stress (118). This may predispose CIH-exposed young adult males to significant oxidative stress because CIH reduces physiological testosterone (92). Testosterone deficiency induces decreased NO bioavailability and endothelial dysfunction, which is restored by testosterone replacement (113, 114, 120). The recovery of endothelial stimulation of NO production with testosterone replacement may occur via the action of estrogen derived from aromatized testosterone (31, 60, 113, 120).

In terms of sympathetic activity, men exhibit higher baseline activity compared with age-matched women, with postmenopausal women showing a greater baseline activity than premenopausal women (121, 122). In addition, essential hypertension in men is associated with a greater level of sympathetic hyperactivity than in age- and body mass index (BMI)-matched women (123). The intrinsic sex differences may underly the exaggerated MAP seen in men during exposures to intermittent hypoxia (121, 122). These reports suggest potential involvement of testosterone in exacerbating sympathetic nervous system activity, which may contribute to CIH hypertension probably via actions on central sympathetic control centers and enhancement of peripheral chemoreflex sensitivity (121–123). However, more research is needed to identify the actions and precise mechanisms of testosterone on sympathetic activity.

**Figure 1.**
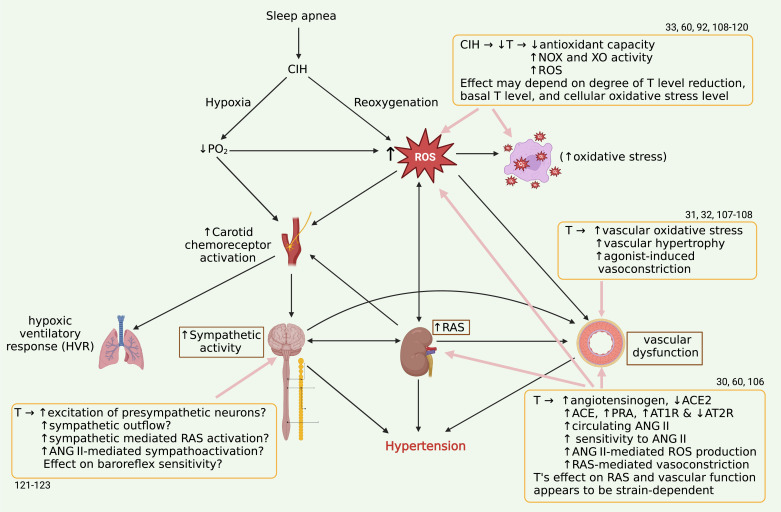
Effects of testosterone (T) on the mechanisms of CIH hypertension. CIH generates hypoxia and reoxygenations leading to increased oxidative stress, generation of RAS-mediated angiotensin II, vascular dysfunction, and autonomic imbalance that leads to hypertension. Bold arrows show pathophysiological mechanisms driving CIH hypertension in an intersystem (CNS, renal, cardiovascular, and respiratory) interplay. Testosterone’s effects on CIH hypertension are shown in the boxes. Created with BioRender. CIH, chronic intermittent hypoxia; CNS, central nervous system; RAS, renin-angiotensin system. The numbers indicate references.

Collectively, the actions of testosterone on vasoconstriction, sympathetic activation, renin-angiotensin system activation, and oxidant mechanisms may influence CIH hypertension. Even though testosterone may contribute to organ system-specific protection depending on strain, it remains to be determined if the combined impact of CIH overrides intrinsic androgenic protections to elicit hypertension in males. Experiments using gonadectomy and hormone replacement are warranted to directly demonstrate whether testosterone is necessary for the development of hypertension, or it merely modulates dysfunction associated with CIH. Elucidating these specific molecular pathways may deepen our understanding of the complex interactions of testosterone with intermittent hypoxia. Figure 1 illustrates how CIH may interact with testosterone (T).

## IMPACT OF ESTROGEN AND PROGESTERONE

### Estrogen and Progesterone Protect against CIH-Induced Oxidative Stress and Ventilatory Instability

Reports on the precise impact of CIH on cycling-induced fluctuations and ovarian hormone levels in gonadally intact females are limited. However, the protective effects of ovarian hormones against CIH hypertension are well-documented and likely multifactorial. Estradiol replacement prevented MAP rise and chemoreflex dysfunction in ovariectomized female Sprague-Dawley rats exposed to 7 days of moderately severe CIH (34, 83).

Estrogen replacement enhanced the antioxidant activity of glutathione peroxidase and superoxide dismutase in the cortex, downregulated pro-oxidant NOX and xanthine oxidase (XO) activity in the brain and adrenal glands, and decreased adrenal protein oxidation in CIH-treated ovariectomized female Sprague-Dawley rats (34). In addition, estrogen and progesterone normalized ROS generation and prevented brain mitochondrial dysfunction in ovariectomized female rodents exposed to CIH (124–126). Many clinical studies involving hormone replacement therapy in postmenopausal women showed a significant reduction in oxidative stress buttressing the potent antioxidant action of estrogen (127–129). Like testosterone, estrogen exerts antioxidant and pro-oxidant effects dependent on cellular oxidative stress status (60, 118, 119). However, its antioxidant capacity could be quenched in unhealthy cells with excessive basal oxidative stress level (60, 118, 119).

Further studies in ovariectomized animals indicate ovarian hormones influence the stabilization of hypoxic ventilatory responses (130, 131). Ovarian hormones decrease the frequency of apneas and increase hypoxic chemosensitivity in CIH-treated rats thereby optimizing respiratory function and mitigating the impact of hypoxia (126, 130, 132). This improves oxygenation and minimizes additive effects of hypoxemia on sympathetic activation to diminish CIH hypertension.

### Impact of Estrogen and Progesterone on Central Autonomic Activity, Renin-Angiotensin System, and Vascular Function

In the proestrus phase of estrus cycle, which is associated with the highest increase in ovarian hormones, intact females show a maximal increase in baroreflex regulation of renal sympathetic nerve activity compared with estrus and diestrus phases but ovariectomy eliminates such variation (131). This report harmonizes with clinical studies that showed that long-term oral and transdermal estrogen replacement therapy improves baroreflex sensitivity and attenuates vascular sympathetic activity in postmenopausal women (133, 134).

Animal studies have identified estrogen receptor (ER) pathways, particularly ERα, as key mediators of cardiovascular protection against intermittent hypoxia exposures (125). Selective ERα but not ERβ agonism prevents the development of hypertension from CIH in ovariectomized rodents (125). Molecular knockdown of ER signaling centrally leads to heightened arterial pressure even in reproductively intact females (135). Conversely, directly stimulating ERα or ERβ expression within autonomic brain regions (paraventricular nucleus, rostral ventrolateral medulla) opposes activation of neurohormonal hypertensive pathways in ovariectomized models (135). The evidence collectively indicates estrogen interaction with central ERs prevents neural overexcitation within preautonomic cardioregulatory sites. Attenuation of glutamatergic excitation by estrogen-ER binding may promote the antihypertensive benefits against heightened sympathetic outflow induced by CIH exposures (36). These observations reveal that estrogen via its action on central ER prevents exaggerated neural excitation in preautonomic nuclei to abrogate CIH-mediated sympathetic and neuroendocrine overactivity.

Estrogen deficiency resulting from ovariectomy leads to central and peripheral upregulation of AT1R expression and increased angiotensin II-mediated vasoconstriction (35, 136, 137). Replacement of the estrogen abolishes these effects and reduces circulating angiotensin II levels while increasing the counterregulatory peptide angiotensin 1–7 (35, 136–138). Similarly, estrogen treatment augments NO bioavailability, stimulating endothelial NO synthase to improve endothelial function and offset vascular oxidative damage and cardiac dysfunction induced by intermittent hypoxia (83, 139, 140). Separately, progesterone blunts norepinephrine-induced pressor effect and exerts an endothelium-independent vasorelaxation by inhibiting calcium influx into vascular smooth muscle (141). The collective impact of enhanced NO-dependent vasodilation and reduced norepinephrine sensitivity contributes to the cardioprotective effects of ovarian hormones.

**Figure 2.**
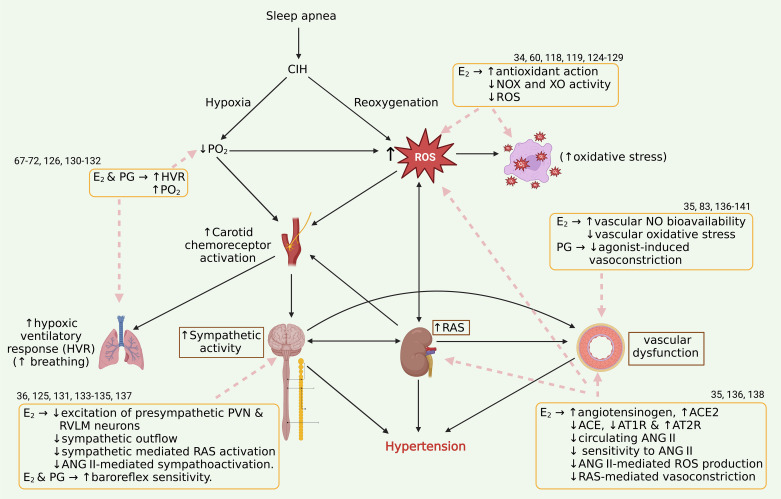
Ovarian hormones (estrogen and progesterone) protect intact/premenopausal females from CIH hypertension by interfering with ROS generation and oxidative stress, preserving vascular function, attenuating angiotensin II (ANG II) effects and exaggerated sympathoactivation. Bold arrows illustrate pathophysiological mechanisms driving CIH hypertension in an intersystem (CNS, renal, cardiovascular, and respiratory) interplay. Dashed arrows show the interferences of estrogen (E_2_) and progesterone (PG) on the mechanisms of CIH to protect intact/premenopausal females from CIH hypertension. Created with BioRender. CIH, chronic intermittent hypoxia; CNS, central nervous system; ROS, reactive oxygen species. The numbers indicate references.

Altogether, by counteracting mechanisms of sympathoexcitation, oxidative stress, and vasoconstriction, estrogen and progesterone confer pleiotropic protective actions against maladaptive cardiovascular changes in CIH-exposed females and several different forms of hypertension. These findings are summarized in Fig. 2.

### Clinical Implications, Perspectives, and Future Direction

Continuous positive airway pressure (CPAP) therapy remains the gold standard treatment for OSA (142). Although hormone replacement therapy is indicated for patients with OSA with hormone deficiencies, such as postmenopausal syndrome, it is not commonly used in patients with hypertensive OSA. This is mainly due to adverse effects such as increased risk of cardiovascular complications (143–146). The challenges associated with OSA treatment extend beyond the limited treatment options currently available to patients. The sex-based differences in disease presentation pose a unique challenge to the diagnosis of OSA especially in premenopausal women. Because premenopausal women with OSA typically present with depression, anxiety, and reduced mental performance and do not appear to develop the cardiovascular phenotype commonly seen in men, the disease can be missed or misdiagnosed. As such, premenopausal women with OSA may be at additional risk of neurocognitive decline due to underdiagnosis (58, 147). Existing animal studies on CIH-induced cognitive impairment have primarily used males, leaving a gap in our understanding of how females are affected. Future studies that investigate sex-based differences in how neuroinflammation, genetic alterations, oxidative stress, and neurodegeneration contribute to CIH-induced neurocognitive decline are needed to address this gap.

To address the sex-based disparities in the detection and management of OSA, early polysomnographic screening in at-risk women with or without hypertension, active detection of OSA in atypically symptomatic women, patient education, and creation of public awareness on the benefits of early diagnosis of OSA and treatment may be beneficial. It is important to standardize approaches for screening symptoms that consider issues such as underreporting of symptoms in women. Implementing standardized approaches that are sensitive to these concerns may resolve these gaps.

Many unresolved questions remain that could unlock opportunities for health-impacting research in CIH and OSA hypertension. The complex interactions between testosterone, aging, and genetic background factors in the context of OSA remain to be fully elucidated. It is still unclear whether sex chromosomes exert effects on CIH-induced hypertension independent of gonadal hormones. The four-core genotype model that allows segregation of chromosomal from hormonal influences on phenotypes (148–153) may be extremely useful for delineating whether genotype differences at the chromosome level impact susceptibility to CIH-hypertension beyond circulating sex steroid mediation. Defining the neurohumoral underpinnings of these sex differences may unveil novel therapeutic targets and strategies to mitigate OSA cardiovascular morbidity in a sex-specific manner. Further clinical studies are warranted to extend these experimental observations to humans and specifically address sex differences in OSA hypertension.

## GRANTS

This review was supported by National Heart, Lung, and Blood Institute Grant R01 HL155977 (to J.T.C., G.E.F., and R.L.C.) and National Institute of Neurological Disorders and Stroke Grant R01 NS0091359 (to R.L.C.).

## DISCLOSURES

No conflicts of interest, financial or otherwise, are declared by the authors.

## AUTHOR CONTRIBUTIONS

C.B.A. prepared figures; C.B.A. and J.T.C. drafted manuscript; C.B.A., J.J.G., G.E.F., R.L.C., and J.T.C. edited and revised manuscript; C.B.A., J.J.G., G.E.F., R.L.C., and J.T.C. approved final version of manuscript.

## References

[1] Ruehland WR, Rochford PD, O'Donoghue FJ, Pierce RJ, Singh P, Thornton AT. The new AASM criteria for scoring hypopneas: impact on the apnea hypopnea index. Sleep 32: 150–157, 2009. doi:10.1093/sleep/32.2.150. 19238801 PMC2635578

[2] Gao Y, Guo Y, Dong J, Liu Y, Hu W, Lu M, Shen Y, Liu Y, Wei Y, Wang Z, Zhan X. Differences in the prevalence of cardiovascular and metabolic diseases coinciding with clinical subtypes of obstructive sleep apnea. Clin Cardiol 46: 92–99, 2023. doi:10.1002/clc.23941. 36403266 PMC9849430

[3] Guo WB, Liu YP, Xu HH, Meng LL, Zhu HM, Wu HM, Guan J, Yi HL, Yin SK. [Obstructive sleep apnea and metabolic syndrome: an association study based on a large sample clinical database]. Zhonghua Er Bi Yan Hou Tou Jing Wai Ke Za Zhi 56: 1263–1269, 2021. doi:10.3760/cma.j.cn115330-20210531-00314. 34963213

[4] Kim T, Kang J. Relationship between obstructive sleep apnea, insulin resistance, and metabolic syndrome: a nationwide population-based survey. Endocr J 70: 107–119, 2023. doi:10.1507/endocrj.EJ22-0280. 36171092

[5] Su X, Han J, Gao Y, He Z, Zhao Z, Lin J, Guo J, Chen K, Gao Y, Liu L. [Correlation of obstructive sleep apnea with components of metabolic syndrome and implications for long-term adverse cardiovascular risk in elderly patients]. Nan Fang Yi Ke Da Xue Xue Bao 41: 1592–1599, 2021. doi:10.12122/j.issn.1673-4254.2021.11.01. 34916183 PMC8685693

[6] Benjafield AV, Ayas NT, Eastwood PR, Heinzer R, Ip MSM, Morrell MJ, Nunez CM, Patel SR, Penzel T, Pepin JL, Peppard PE, Sinha S, Tufik S, Valentine K, Malhotra A. Estimation of the global prevalence and burden of obstructive sleep apnoea: a literature-based analysis. Lancet Respir Med 7: 687–698, 2019. doi:10.1016/S2213-2600(19)30198-5. 31300334 PMC7007763

[7] Peppard PE, Young T, Barnet JH, Palta M, Hagen EW, Hla KM. Increased prevalence of sleep-disordered breathing in adults. Am J Epidemiol 177: 1006–1014, 2013. doi:10.1093/aje/kws342. 23589584 PMC3639722

[8] Franklin KA, Lindberg E. Obstructive sleep apnea is a common disorder in the population-a review on the epidemiology of sleep apnea. J Thorac Dis 7: 1311–1322, 2015. doi:10.3978/j.issn.2072-1439.2015.06.11. 26380759 PMC4561280

[9] Lin CM, Davidson TM, Ancoli-Israel S. Gender differences in obstructive sleep apnea and treatment implications. Sleep Med Rev 12: 481–496, 2008. doi:10.1016/j.smrv.2007.11.003. 18951050 PMC2642982

[10] Wimms A, Woehrle H, Ketheeswaran S, Ramanan D, Armitstead J. Obstructive sleep apnea in women: specific issues and interventions. Biomed Res Int 2016: 1764837, 2016. doi:10.1155/2016/1764837. 27699167 PMC5028797

[11] Geer JH, Hilbert J. Gender issues in obstructive sleep apnea. Yale J Biol Med 94: 487–496, 2021. 34602886 PMC8461585

[12] Kario K. Obstructive sleep apnea syndrome and hypertension: mechanism of the linkage and 24-h blood pressure control. Hypertens Res 32: 537–541, 2009. doi:10.1038/hr.2009.73. 19461649

[13] Yang C, Zhou Y, Liu H, Xu P. The role of inflammation in cognitive impairment of obstructive sleep apnea syndrome. Brain Sci 12: 1303, 2022. doi:10.3390/brainsci12101303. 36291237 PMC9599901

[14] Seda G, Matwiyoff G, Parrish JS. Effects of obstructive sleep apnea and CPAP on cognitive function. Curr Neurol Neurosci Rep 21: 32, 2021. doi:10.1007/s11910-021-01123-0. 33956247

[15] Sharifpour P, Dehvan F, Dalvand S, Ghanei Gheshlagh R. Examination of the relationship between metabolic syndrome and obstructive sleep apnea in Iranian patients with type 2 diabetes: a case-control study. Diabetes Metab Syndr Obes 13: 2251–2257, 2020. doi:10.2147/DMSO.S260677. 32617014 PMC7326211

[16] Ernst G, Bosio M, Salvado A, Dibur E, Nigro C, Borsini E. Difference between apnea-hypopnea index (AHI) and oxygen desaturation index (ODI): proportional increase associated with degree of obesity. Sleep Breath 20: 1175–1183, 2016. doi:10.1007/s11325-016-1330-3. 27026417

[17] Votteler S, Knaack L, Janicki J, Fink GR, Burghaus L. Sex differences in polysomnographic findings in patients with obstructive sleep apnea. Sleep Med 101: 429–436, 2023. doi:10.1016/j.sleep.2022.11.025. 36516599

[18] Wahner-Roedler DL, Olson EJ, Narayanan S, Sood R, Hanson AC, Loehrer LL, Sood A. Gender-specific differences in a patient population with obstructive sleep apnea-hypopnea syndrome. Gend Med 4: 329–338, 2007. doi:10.1016/s1550-8579(07)80062-3. 18215724

[19] Honig E, Green A, Dagan Y. Gender differences in the sleep variables contributing to excessive daytime sleepiness among patients with obstructive sleep apnea. Sleep Breath 25: 1837–1842, 2021. doi:10.1007/s11325-020-02276-x. 33464468 PMC8590667

[20] Chen LD, Huang JF, Chen GP, Zeng AM, Chen MX, Chen ML, Lin QC. [Association and gender difference analysis of obstructive sleep apnea hypopnea syndrome and liver injury]. Zhonghua Yi Xue Za Zhi 102: 550–554, 2022. doi:10.3760/cma.j.cn112137-20210617-01371. 35196776

[21] Tancic-Gajic M, Vukcevic M, Ivovic M, Marina LV, Arizanovic Z, Soldatovic I, Stojanovic M, Dogo A, Kendereski A, Vujovic S. Obstructive sleep apnea is associated with low testosterone levels in severely obese men. Front Endocrinol (Lausanne) 12: 622496, 2021. doi:10.3389/fendo.2021.622496. 34381420 PMC8350060

[22] Leppanen T, Kulkas A, Duce B, Mervaala E, Toyras J. Severity of individual obstruction events is gender dependent in sleep apnea. Sleep Breath 21: 397–404, 2017. doi:10.1007/s11325-016-1430-0. 27966055

[23] Gabbay IE, Lavie P. Age- and gender-related characteristics of obstructive sleep apnea. Sleep Breath 16: 453–460, 2012. doi:10.1007/s11325-011-0523-z. 21499842

[24] Sigurðardóttir ES, Gislason T, Benediktsdottir B, Hustad S, Dadvand P, Demoly P, Franklin KA, Heinrich J, Holm M, van der Plaat DA, Jõgi R, Leynaert B, Lindberg E, Martinez-Moratalla J, Sainz De Aja L, Pesce G, Pin I, Raherison C, Pereira-Vega A, Real FG, Triebner K. Female sex hormones and symptoms of obstructive sleep apnea in European women of a population-based cohort. PLoS One 17: e0269569, 2022. doi:10.1371/journal.pone.0269569. 35731786 PMC9216532

[25] Schiza SE, Bouloukaki I. Does gender matter: sex-specific aspects of symptoms, outcome, and therapy of obstructive sleep apnea. Curr Opin Pulm Med 26: 642–649, 2020. doi:10.1097/MCP.0000000000000728. 32890020

[26] Buratti L, Rocchi C, Totaro V, Broggi S, Lattanzi S, Viticchi G, Falsetti L, Silvestrini M. Sex-related differences in polygraphic parameters in a population of patients with obstructive sleep apnea syndrome. CNS Neurol Disord Drug Targets 21: 492–499, 2022. doi:10.2174/1871527320666211022104140. 34719367

[27] Cano-Pumarega I, Barbe F, Esteban A, Martinez-Alonso M, Egea C, Duran-Cantolla J; Spanish Sleep Network. Sleep apnea and hypertension: are there sex differences? The Vitoria Sleep Cohort. Chest 152: 742–750, 2017. doi:10.1016/j.chest.2017.03.008. 28300571

[28] Knight WD, Little JT, Carreno FR, Toney GM, Mifflin SW, Cunningham JT. Chronic intermittent hypoxia increases blood pressure and expression of FosB/DeltaFosB in central autonomic regions. Am J Physiol Regul Integr Comp Physiol 301: R131–R139, 2011. doi:10.1152/ajpregu.00830.2010. 21543638 PMC3129875

[29] Fletcher EC. Invited review: Physiological consequences of intermittent hypoxia: systemic blood pressure. J Appl Physiol (1985) 90: 1600–1605, 2001. doi:10.1152/jappl.2001.90.4.1600. 11247966

[30] Chen YF, Naftilan AJ, Oparil S. Androgen-dependent angiotensinogen and renin messenger RNA expression in hypertensive rats. Hypertension 19: 456–463, 1992. doi:10.1161/01.hyp.19.5.456. 1568764

[31] Chignalia AZ, Schuldt EZ, Camargo LL, Montezano AC, Callera GE, Laurindo FR, Lopes LR, Avellar MC, Carvalho MH, Fortes ZB, Touyz RM, Tostes RC. Testosterone induces vascular smooth muscle cell migration by NADPH oxidase and c-Src-dependent pathways. Hypertension 59: 1263–1271, 2012. doi:10.1161/HYPERTENSIONAHA.111.180620. 22566500

[32] Song J, Kost CK Jr, Martin DS. Androgens potentiate renal vascular responses to angiotensin II via amplification of the Rho kinase signaling pathway. Cardiovasc Res 72: 456–463, 2006. doi:10.1016/j.cardiores.2006.09.007. 17049502

[33] Tenkorang MAA, Duong P, Cunningham RL. NADPH oxidase mediates membrane androgen receptor-induced neurodegeneration. Endocrinology 160: 947–963, 2019. doi:10.1210/en.2018-01079. 30811529 PMC6435014

[34] Laouafa S, Ribon-Demars A, Marcouiller F, Roussel D, Bairam A, Pialoux V, Joseph V. Estradiol protects against cardiorespiratory dysfunctions and oxidative stress in intermittent hypoxia. Sleep 40: zsx104, 2017 [Erratum in Sleep 43: zsaa025]. doi:10.1093/sleep/zsx104. 28633495

[35] Dean SA, Tan J, O'Brien ER, Leenen FH. 17beta-estradiol downregulates tissue angiotensin-converting enzyme and ANG II type 1 receptor in female rats. Am J Physiol Regul Integr Comp Physiol 288: R759–R766, 2005. doi:10.1152/ajpregu.00595.2004. 15550614

[36] Gingerich S, Krukoff TL. Estrogen in the paraventricular nucleus attenuates L-glutamate-induced increases in mean arterial pressure through estrogen receptor beta and NO. Hypertension 48: 1130–1136, 2006. doi:10.1161/01.HYP.0000248754.67128.ff. 17075034

[37] Navarrete-Opazo A, Mitchell GS. Therapeutic potential of intermittent hypoxia: a matter of dose. Am J Physiol Regul Integr Comp Physiol 307: R1181–R1197, 2014. doi:10.1152/ajpregu.00208.2014. 25231353 PMC4315448

[38] Fletcher EC, Lesske J, Behm R, Miller CC 3rd, Stauss H, Unger T. Carotid chemoreceptors, systemic blood pressure, and chronic episodic hypoxia mimicking sleep apnea. J Appl Physiol (1985) 72: 1978–1984, 1992. doi:10.1152/jappl.1992.72.5.1978. 1601808

[39] Fletcher EC, Bao G, Li R. Renin activity and blood pressure in response to chronic episodic hypoxia. Hypertension 34: 309–314, 1999. doi:10.1161/01.hyp.34.2.309. 10454459

[40] Lesske J, Fletcher EC, Bao G, Unger T. Hypertension caused by chronic intermittent hypoxia–influence of chemoreceptors and sympathetic nervous system. J Hypertens 15: 1593–1603, 1997. doi:10.1097/00004872-199715120-00060. 9488210

[41] Snyder B, Duong P, Tenkorang M, Wilson EN, Cunningham RL. Rat strain and housing conditions alter oxidative stress and hormone responses to chronic intermittent hypoxia. Front Physiol 9: 1554, 2018. doi:10.3389/fphys.2018.01554. 30459637 PMC6232418

[42] Greenberg HE, Sica A, Batson D, Scharf SM. Chronic intermittent hypoxia increases sympathetic responsiveness to hypoxia and hypercapnia. J Appl Physiol (1985) 86: 298–305, 1999. doi:10.1152/jappl.1999.86.1.298. 9887143

[43] Dick TE, Hsieh YH, Wang N, Prabhakar N. Acute intermittent hypoxia increases both phrenic and sympathetic nerve activities in the rat. Exp Physiol 92: 87–97, 2007. doi:10.1113/expphysiol.2006.035758. 17138622

[44] Del Rio R, Moya EA, Iturriaga R. Carotid body potentiation during chronic intermittent hypoxia: implication for hypertension. Front Physiol 5: 434, 2014. doi:10.3389/fphys.2014.00434. 25429271 PMC4228839

[45] Lam SY, Liu Y, Ng KM, Liong EC, Tipoe GL, Leung PS, Fung ML. Upregulation of a local renin-angiotensin system in the rat carotid body during chronic intermittent hypoxia. Exp Physiol 99: 220–231, 2014. doi:10.1113/expphysiol.2013.074591. 24036592

[46] Tahawi Z, Orolinova N, Joshua IG, Bader M, Fletcher EC. Altered vascular reactivity in arterioles of chronic intermittent hypoxic rats. J Appl Physiol (1985) 90: 2007–2013, 2001. doi:10.1152/jappl.2001.90.5.2007. 11299297

[47] Del Rio R, Andrade DC, Lucero C, Arias P, Iturriaga R. Carotid body ablation abrogates hypertension and autonomic alterations induced by intermittent hypoxia in rats. Hypertension 68: 436–445, 2016. doi:10.1161/HYPERTENSIONAHA.116.07255. 27381902

[48] Phillips SA, Olson EB, Morgan BJ, Lombard JH. Chronic intermittent hypoxia impairs endothelium-dependent dilation in rat cerebral and skeletal muscle resistance arteries. Am J Physiol Heart Circ Physiol 286: H388–H393, 2004. doi:10.1152/ajpheart.00683.2003. 14512283

[49] Cunningham JT, Knight WD, Mifflin SW, Nestler EJ. An Essential role for DeltaFosB in the median preoptic nucleus in the sustained hypertensive effects of chronic intermittent hypoxia. Hypertension 60: 179–187, 2012. doi:10.1161/HYPERTENSIONAHA.112.193789. 22689746 PMC3415378

[50] Knight WD, Saxena A, Shell B, Nedungadi TP, Mifflin SW, Cunningham JT. Central losartan attenuates increases in arterial pressure and expression of FosB/DeltaFosB along the autonomic axis associated with chronic intermittent hypoxia. Am J Physiol Regul Integr Comp Physiol 305: R1051–R1058, 2013. doi:10.1152/ajpregu.00541.2012. 24026072 PMC3840317

[51] Hinojosa-Laborde C, Mifflin SW. Sex differences in blood pressure response to intermittent hypoxia in rats. Hypertension 46: 1016–1021, 2005. doi:10.1161/01.HYP.0000175477.33816.f3. 16157795

[52] Prabhakar NR, Kumar GK, Nanduri J, Semenza GL. ROS signaling in systemic and cellular responses to chronic intermittent hypoxia. Antioxid Redox Signal 9: 1397–1403, 2007. doi:10.1089/ars.2007.1732. 17627465

[53] Troncoso Brindeiro CM, da Silva AQ, Allahdadi KJ, Youngblood V, Kanagy NL. Reactive oxygen species contribute to sleep apnea-induced hypertension in rats. Am J Physiol Heart Circ Physiol 293: H2971–H2976, 2007. doi:10.1152/ajpheart.00219.2007. 17766485 PMC3792788

[54] Lam SY, Liu Y, Ng KM, Lau CF, Liong EC, Tipoe GL, Fung ML. Chronic intermittent hypoxia induces local inflammation of the rat carotid body via functional upregulation of proinflammatory cytokine pathways. Histochem Cell Biol 137: 303–317, 2012. doi:10.1007/s00418-011-0900-5. 22187044 PMC3278607

[55] Kapsimalis F, Kryger MH. Gender and obstructive sleep apnea syndrome, part 1: clinical features. Sleep 25: 412–419, 2002. 12071542

[56] Levartovsky A, Dafna E, Zigel Y, Tarasiuk A. Breathing and snoring sound characteristics during sleep in adults. J Clin Sleep Med 12: 375–384, 2016. doi:10.5664/jcsm.5588. 26518701 PMC4773633

[57] Sforza E, Chouchou F, Collet P, Pichot V, Barthelemy JC, Roche F. Sex differences in obstructive sleep apnoea in an elderly French population. Eur Respir J 37: 1137–1143, 2011. doi:10.1183/09031936.00043210. 20817711

[58] Sjosten N, Vahtera J, Salo P, Oksanen T, Saaresranta T, Virtanen M, Pentti J, Kivimaki M. Increased risk of lost workdays prior to the diagnosis of sleep apnea. Chest 136: 130–136, 2009. doi:10.1378/chest.08-2201. 19318680

[59] Cai LQ, Huang L, Wei LL, Yao JS, Xu LY, Chen W. Reduced plasma estradiol levels are associated with sleep apnea in depressed peri- and post-menopausal women. Neuropsychiatr Dis Treat 17: 3483–3488, 2021. doi:10.2147/NDT.S333154. 34880617 PMC8648326

[60] Sumien N, Cunningham JT, Davis DL, Engelland R, Fadeyibi O, Farmer GE, Mabry S, Mensah-Kane P, Trinh OTP, Vann PH, Wilson EN, Cunningham RL. Neurodegenerative disease: roles for sex, hormones, and oxidative stress. Endocrinology 162: bqab185, 2021. doi:10.1210/endocr/bqab185. 34467976 PMC8462383

[61] Bourgonje AR, Abdulle AE, Al-Rawas AM, Al-Maqbali M, Al-Saleh M, Enriquez MB, Al-Siyabi S, Al-Hashmi K, Al-Lawati I, Bulthuis MLC, Mulder DJ, Gordijn SJ, van Goor H, Saleh J. Systemic oxidative stress is increased in postmenopausal women and independently associates with homocysteine levels. Int J Mol Sci 21: 314, 2020. doi:10.3390/ijms21010314. 31906485 PMC6982320

[62] Kim SD, Cho KS. Obstructive sleep apnea and testosterone deficiency. World J Mens Health 37: 12–18, 2019. doi:10.5534/wjmh.180017. 29774669 PMC6305865

[63] Stavaras C, Pastaka C, Papala M, Gravas S, Tzortzis V, Melekos M, Seitanidis G, Gourgoulianis KI. Sexual function in pre- and post-menopausal women with obstructive sleep apnea syndrome. Int J Impot Res 24: 228–233, 2012. doi:10.1038/ijir.2012.20. 22673583

[64] Mielke MM, Kapoor E, Geske JR, Fields JA, LeBrasseur NK, Morrow MM, Winham SJ, Faubion LL, Castillo AM, Hofrenning EI, Bailey KR, Rocca WA, Kantarci K. Long-term effects of premenopausal bilateral oophorectomy with or without hysterectomy on physical aging and chronic medical conditions. Menopause 30: 1090–1097, 2023. doi:10.1097/GME.0000000000002254. 37699239 PMC10615715

[65] Shuster LT, Gostout BS, Grossardt BR, Rocca WA. Prophylactic oophorectomy in premenopausal women and long-term health. Menopause Int 14: 111–116, 2008. doi:10.1258/mi.2008.008016. 18714076 PMC2585770

[66] Kapoor E, Faubion SS, Gazzuola Rocca L, Mielke MM, Smith CY, Rocca WA. Trajectories of metabolic parameters after bilateral oophorectomy in premenopausal women. Maturitas 165: 38–46, 2022. doi:10.1016/j.maturitas.2022.07.005. 35905571 PMC9529838

[67] Keefe DL, Watson R, Naftolin F. Hormone replacement therapy may alleviate sleep apnea in menopausal women: a pilot study. Menopause 6: 196–200, 1999. doi:10.1097/00042192-199906030-00004. 10486788

[68] Manber R, Kuo TF, Cataldo N, Colrain IM. The effects of hormone replacement therapy on sleep-disordered breathing in postmenopausal women: a pilot study. Sleep 26: 163–168, 2003. 12683475

[69] Pickett CK, Regensteiner JG, Woodard WD, Hagerman DD, Weil JV, Moore LG. Progestin and estrogen reduce sleep-disordered breathing in postmenopausal women. J Appl Physiol (1985) 66: 1656–1661, 1989. doi:10.1152/jappl.1989.66.4.1656. 2543656

[70] Wesstrom J, Ulfberg J, Nilsson S. Sleep apnea and hormone replacement therapy: a pilot study and a literature review. Acta Obstet Gynecol Scand 84: 54–57, 2005. doi:10.1111/j.0001-6349.2005.00575.x. 15603568

[71] Dancey DR, Hanly PJ, Soong C, Lee B, Hoffstein V. Impact of menopause on the prevalence and severity of sleep apnea. Chest 120: 151–155, 2001. doi:10.1378/chest.120.1.151. 11451831

[72] Regensteiner JG, Woodard WD, Hagerman DD, Weil JV, Pickett CK, Bender PR, Moore LG. Combined effects of female hormones and metabolic rate on ventilatory drives in women. J Appl Physiol (1985) 66: 808–813, 1989. doi:10.1152/jappl.1989.66.2.808. 2540141

[73] Kim J, Freeman K, Ayala A, Mullen M, Sun Z, Rhee JW. Cardiovascular impact of androgen deprivation therapy: from basic biology to clinical practice. Curr Oncol Rep 25: 965–977, 2023. doi:10.1007/s11912-023-01424-2. 37273124 PMC10474986

[74] Huddart RA, Norman A, Moynihan C, Horwich A, Parker C, Nicholls E, Dearnaley DP. Fertility, gonadal and sexual function in survivors of testicular cancer. Br J Cancer 93: 200–207, 2005. doi:10.1038/sj.bjc.6602677. 15999104 PMC2361550

[75] Smith JC, Bennett S, Evans LM, Kynaston HG, Parmar M, Mason MD, Cockcroft JR, Scanlon MF, Davies JS. The effects of induced hypogonadism on arterial stiffness, body composition, and metabolic parameters in males with prostate cancer. J Clin Endocrinol Metab 86: 4261–4267, 2001. doi:10.1210/jcem.86.9.7851. 11549659

[76] Hoyos CM, Killick R, Yee BJ, Grunstein RR, Liu PY. Effects of testosterone therapy on sleep and breathing in obese men with severe obstructive sleep apnoea: a randomized placebo-controlled trial. Clin Endocrinol (Oxf) 77: 599–607, 2012. doi:10.1111/j.1365-2265.2012.04413.x. 22512435

[77] Hanafy HM. Testosterone therapy and obstructive sleep apnea: is there a real connection? J Sex Med 4: 1241–1246, 2007. doi:10.1111/j.1743-6109.2007.00553.x. 17645445

[78] Cole AP, Hanske J, Jiang W, Kwon NK, Lipsitz SR, Kathrins M, Learn PA, Sun M, Haider AH, Basaria S, Trinh QD. Impact of testosterone replacement therapy on thromboembolism, heart disease and obstructive sleep apnoea in men. BJU Int 121: 811–818, 2018. doi:10.1111/bju.14149. 29383868

[79] Young T, Finn L, Peppard PE, Szklo-Coxe M, Austin D, Nieto FJ, Stubbs R, Hla KM. Sleep disordered breathing and mortality: eighteen-year follow-up of the Wisconsin sleep cohort. Sleep 31: 1071–1078, 2008. 18714778 PMC2542952

[80] Young T, Peppard P, Palta M, Hla KM, Finn L, Morgan B, Skatrud J. Population-based study of sleep-disordered breathing as a risk factor for hypertension. Arch Intern Med 157: 1746–1752, 1997. 9250236

[81] Nieto FJ, Young TB, Lind BK, Shahar E, Samet JM, Redline S, D'Agostino RB, Newman AB, Lebowitz MD, Pickering TG. Association of sleep-disordered breathing, sleep apnea, and hypertension in a large community-based study. Sleep Heart Health Study. JAMA 283: 1829–1836, 2000 [Erratum in JAMA 288: 1985, 2002]. doi:10.1001/jama.283.14.1829. 10770144

[82] Souza GM, Bonagamba LG, Amorim MR, Moraes DJ, Machado BH. Cardiovascular and respiratory responses to chronic intermittent hypoxia in adult female rats. Exp Physiol 100: 249–258, 2015. doi:10.1113/expphysiol.2014.082990. 25631702

[83] Ribon-Demars A, Pialoux V, Boreau A, Marcouiller F, Lariviere R, Bairam A, Joseph V. Protective roles of estradiol against vascular oxidative stress in ovariectomized female rats exposed to normoxia or intermittent hypoxia. Acta Physiol (Oxf) 225: e13159, 2019. doi:10.1111/apha.13159. 29947475

[84] Ayala-Mendez GX, Calderon VM, Zuniga-Pimentel TA, Rivera-Cerecedo CV. Noninvasive monitoring of blood pressure and heart rate during estrous cycle phases in normotensive Wistar-Kyoto and spontaneously hypertensive female rats. J Am Assoc Lab Anim Sci 62: 267–273, 2023. doi:10.30802/AALAS-JAALAS-22-000081. 37130700 PMC10230531

[85] Grundt A, Grundt C, Knoth K, Lemmer B. Rhythmic supplementation of 17beta-estradiol according to the physiological estrous cycle: effect on blood pressure in female ovariectomized rats. Horm Metab Res 42: 130–136, 2010. doi:10.1055/s-0029-1241807. 19882501

[86] Loh SY, Salleh N. Influence of testosterone on mean arterial pressure: A physiological study in male and female normotensive WKY and hypertensive SHR rats. Physiol Int 104: 25–34, 2017. doi:10.1556/2060.104.2017.1.3. 28361574

[87] Capone C, Anrather J, Milner TA, Iadecola C. Estrous cycle-dependent neurovascular dysfunction induced by angiotensin II in the mouse neocortex. Hypertension 54: 302–307, 2009. doi:10.1161/HYPERTENSIONAHA.109.133249. 19506098 PMC2750855

[88] Ely D, Milsted A, Dunphy G, Boehme S, Dunmire J, Hart M, Toot J, Turner M. Delivery of sry1, but not sry2, to the kidney increases blood pressure and sns indices in normotensive wky rats. BMC Physiol 9: 10, 2009. doi:10.1186/1472-6793-9-10. 19500370 PMC2699329

[89] Ely D, Caplea A, Dunphy G, Daneshvar H, Turner M, Milsted A, Takiyyudin M. Spontaneously hypertensive rat Y chromosome increases indexes of sympathetic nervous system activity. Hypertension 29: 613–618, 1997. doi:10.1161/01.hyp.29.2.613. 9040447

[90] Ji H, Zheng W, Wu X, Liu J, Ecelbarger CM, Watkins R, Arnold AP, Sandberg K. Sex chromosome effects unmasked in angiotensin II-induced hypertension. Hypertension 55: 1275–1282, 2010. doi:10.1161/HYPERTENSIONAHA.109.144949. 20231528 PMC2905778

[91] Sandberg K, Ji H. Sex differences in primary hypertension. Biol Sex Differ 3: 7, 2012. doi:10.1186/2042-6410-3-7. 22417477 PMC3331829

[92] Wilson EN, Anderson M, Snyder B, Duong P, Trieu J, Schreihofer DA, Cunningham RL. Chronic intermittent hypoxia induces hormonal and male sexual behavioral changes: hypoxia as an advancer of aging. Physiol Behav 189: 64–73, 2018. doi:10.1016/j.physbeh.2018.03.007. 29526572 PMC5882542

[93] Cho YM, Chou JC, Fang CM, Hu S, Wang KL, Wang SW, Wang PS. Chronic intermittent hypoxia stimulates testosterone production in rat Leydig cells. Life Sci 233: 116694, 2019. doi:10.1016/j.lfs.2019.116694. 31351970

[94] Hwang GS, Chen ST, Chen TJ, Wang SW. Effects of hypoxia on testosterone release in rat Leydig cells. Am J Physiol Endocrinol Metab 297: E1039–E1045, 2009. doi:10.1152/ajpendo.00010.2009. 19690072

[95] Bosc LV, Resta T, Walker B, Kanagy NL. Mechanisms of intermittent hypoxia induced hypertension. J Cell Mol Med 14: 3–17, 2010. doi:10.1111/j.1582-4934.2009.00929.x. 19818095 PMC3649074

[96] Quintero M, Olea E, Conde SV, Obeso A, Gallego-Martin T, Gonzalez C, Monserrat JM, Gomez-Nino A, Yubero S, Agapito T. Age protects from harmful effects produced by chronic intermittent hypoxia. J Physiol 594: 1773–1790, 2016. doi:10.1113/JP270878. 26752660 PMC4799969

[97] Marciante AB, Wang LA, Little JT, Cunningham JT. Caspase lesions of PVN-projecting MnPO neurons block the sustained component of CIH-induced hypertension in adult male rats. Am J Physiol Heart Circ Physiol 318: H34–H48, 2020. doi:10.1152/ajpheart.00350.2019. 31675258 PMC6985804

[98] Faulk K, Shell B, Nedungadi TP, Cunningham JT. Role of angiotensin-converting enzyme 1 within the median preoptic nucleus following chronic intermittent hypoxia. Am J Physiol Regul Integr Comp Physiol 312: R245–R252, 2017. doi:10.1152/ajpregu.00472.2016. 28003214 PMC5336571

[99] Soukhova-O'Hare GK, Ortines RV, Gu Y, Nozdrachev AD, Prabhu SD, Gozal D. Postnatal intermittent hypoxia and developmental programming of hypertension in spontaneously hypertensive rats: the role of reactive oxygen species and L-Ca^2+^ channels. Hypertension 52: 156–162, 2008. doi:10.1161/HYPERTENSIONAHA.108.110296. 18474836

[100] Soukhova-O'Hare GK, Cheng ZJ, Roberts AM, Gozal D. Postnatal intermittent hypoxia alters baroreflex function in adult rats. Am J Physiol Heart Circ Physiol 290: H1157–H1164, 2006. doi:10.1152/ajpheart.00767.2005. 16155099

[101] Reckelhoff JF, Zhang H, Srivastava K, Granger JP. Gender differences in hypertension in spontaneously hypertensive rats: role of androgens and androgen receptor. Hypertension 34: 920–923, 1999. doi:10.1161/01.hyp.34.4.920. 10523385

[102] Chen YF, Meng QC. Sexual dimorphism of blood pressure in spontaneously hypertensive rats is androgen dependent. Life Sci 48: 85–96, 1991. doi:10.1016/0024-3205(91)90428-e. 1986184

[103] Reckelhoff JF, Zhang H, Granger JP. Testosterone exacerbates hypertension and reduces pressure-natriuresis in male spontaneously hypertensive rats. Hypertension 31: 435–439, 1998. doi:10.1161/01.hyp.31.1.435. 9453341

[104] Ganten U, Schroder G, Witt M, Zimmermann F, Ganten D, Stock G. Sexual dimorphism of blood pressure in spontaneously hypertensive rats: effects of anti-androgen treatment. J Hypertens 7: 721–726, 1989. 2529310

[105] Dalmasso C, Patil CN, Yanes Cardozo LL, Romero DG, Maranon RO. Cardiovascular and metabolic consequences of testosterone supplements in young and old male spontaneously hypertensive rats: implications for testosterone supplements in men. J Am Heart Assoc 6: e007074, 2017. doi:10.1161/jaha.117.007074. 29042425 PMC5721890

[106] Hanson AE, Perusquia M, Stallone JN. Hypogonadal hypertension in male Sprague-Dawley rats is renin-angiotensin system-dependent: role of endogenous androgens. Biol Sex Differ 11: 48, 2020. doi:10.1186/s13293-020-00324-5. 32843085 PMC7448502

[107] Mishra JS, More AS, Gopalakrishnan K, Kumar S. Testosterone plays a permissive role in angiotensin II-induced hypertension and cardiac hypertrophy in male rats. Biol Reprod 100: 139–148, 2019. doi:10.1093/biolre/ioy179. 30102356 PMC6335213

[108] Dantas APV, Franco M. D C P, Silva-Antonialli MM, Tostes RCA, Fortes ZB, Nigro D, Carvalho MHC. Gender differences in superoxide generation in microvessels of hypertensive rats: role of NAD(P)H-oxidase. Cardiovasc Res 61: 22–29, 2004. doi:10.1016/j.cardiores.2003.10.010. 14732198

[109] Ide T, Tsutsui H, Ohashi N, Hayashidani S, Suematsu N, Tsuchihashi M, Tamai H, Takeshita A. Greater oxidative stress in healthy young men compared with premenopausal women. Arterioscler Thromb Vasc Biol 22: 438–442, 2002. doi:10.1161/hq0302.104515. 11884287

[110] Bhatia K, Elmarakby AA, El-Remessy AB, Sullivan JC. Oxidative stress contributes to sex differences in angiotensin II-mediated hypertension in spontaneously hypertensive rats. Am J Physiol Regul Integr Comp Physiol 302: R274–R282, 2012 [Erratum in Am J Physiol Regul Integr Comp Physiol 302: R1233, 2012]. doi:10.1152/ajpregu.00546.2011. 22049231 PMC3349386

[111] Chen Y, Ji L-L, Liu T-Y, Wang Z-T. Evaluation of gender-related differences in various oxidative stress enzymes in mice. Chin J Physiol 54: 385–390, 2011. doi:10.4077/cjp.2011.amm080. 22229505

[112] Barp J, Araujo AS, Fernandes TR, Rigatto KV, Llesuy S, Bello-Klein A, Singal P. Myocardial antioxidant and oxidative stress changes due to sex hormones. Braz J Med Biol Res 35: 1075–1081, 2002. doi:10.1590/s0100-879x2002000900008. 12219179

[113] Hotta Y, Kataoka T, Kimura K. Testosterone deficiency and endothelial dysfunction: nitric oxide, asymmetric dimethylarginine, and endothelial progenitor cells. Sex Med Rev 7: 661–668, 2019. doi:10.1016/j.sxmr.2019.02.005. 30987932

[114] Lopes RA, Neves KB, Carneiro FS, Tostes RC. Testosterone and vascular function in aging. Front Physiol 3: 89, 2012. doi:10.3389/fphys.2012.00089. 22514541 PMC3322529

[115] Snyder B, Duong P, Trieu J, Cunningham RL. Androgens modulate chronic intermittent hypoxia effects on brain and behavior. Horm Behav 106: 62–73, 2018. doi:10.1016/j.yhbeh.2018.09.005. 30268884 PMC6486829

[116] Pongkan W, Chattipakorn SC, Chattipakorn N. Chronic testosterone replacement exerts cardioprotection against cardiac ischemia-reperfusion injury by attenuating mitochondrial dysfunction in testosterone-deprived rats. PLoS One 10: e0122503, 2015. doi:10.1371/journal.pone.0122503. 25822979 PMC4379072

[117] Zhang L, Wu S, Ruan Y, Hong L, Xing X, Lai W. Testosterone suppresses oxidative stress via androgen receptor-independent pathway in murine cardiomyocytes. Mol Med Rep 4: 1183–1188, 2011. doi:10.3892/mmr.2011.539. 21785825

[118] Holmes S, Singh M, Su C, Cunningham RL. Effects of oxidative stress and testosterone on pro-inflammatory signaling in a female rat dopaminergic neuronal cell line. Endocrinology 157: 2824–2835, 2016. doi:10.1210/en.2015-1738. 27167771 PMC4929547

[119] Duong P, Tenkorang MAA, Trieu J, McCuiston C, Rybalchenko N, Cunningham RL. Neuroprotective and neurotoxic outcomes of androgens and estrogens in an oxidative stress environment. Biol Sex Differ 11: 12, 2020. doi:10.1186/s13293-020-0283-1. 32223745 PMC7104511

[120] Yu J, Akishita M, Eto M, Ogawa S, Son BK, Kato S, Ouchi Y, Okabe T. Androgen receptor-dependent activation of endothelial nitric oxide synthase in vascular endothelial cells: role of phosphatidylinositol 3-kinase/akt pathway. Endocrinology 151: 1822–1828, 2010. doi:10.1210/en.2009-1048. 20194727

[121] Ng AV, Callister R, Johnson DG, Seals DR. Age and gender influence muscle sympathetic nerve activity at rest in healthy humans. Hypertension 21: 498–503, 1993. doi:10.1161/01.hyp.21.4.498. 8458648

[122] Jones PP, Davy KP, Seals DR. Influence of gender on the sympathetic neural adjustments to alterations in systemic oxygen levels in humans. Clin Physiol 19: 153–160, 1999. doi:10.1046/j.1365-2281.1999.00158.x. 10200897

[123] Hogarth AJ, Mackintosh AF, Mary DA. The effect of gender on the sympathetic nerve hyperactivity of essential hypertension. J Hum Hypertens 21: 239–245, 2007. doi:10.1038/sj.jhh.1002132. 17167522

[124] Strehlow K, Rotter S, Wassmann S, Adam O, Grohe C, Laufs K, Bohm M, Nickenig G. Modulation of antioxidant enzyme expression and function by estrogen. Circ Res 93: 170–177, 2003. doi:10.1161/01.RES.0000082334.17947.11. 12816884

[125] Laouafa S, Roussel D, Marcouiller F, Soliz J, Gozal D, Bairam A, Joseph V. Roles of oestradiol receptor alpha and beta against hypertension and brain mitochondrial dysfunction under intermittent hypoxia in female rats. Acta Physiol (Oxf) 226: e13255, 2019. doi:10.1111/apha.13255. 30635990 PMC7590630

[126] Joseph V, Laouafa S, Marcouiller F, Roussel D, Pialoux V, Bairam A. Progesterone decreases apnoea and reduces oxidative stress induced by chronic intermittent hypoxia in ovariectomized female rats. Exp Physiol 105: 1025–1034, 2020. doi:10.1113/EP088430. 32196792

[127] Sanchez-Rodriguez MA, Zacarias-Flores M, Castrejon-Delgado L, Ruiz-Rodriguez AK, Mendoza-Nunez VM. Effects of hormone therapy on oxidative stress in postmenopausal women with metabolic syndrome. Int J Mol Sci 17: 1388, 2016. doi:10.3390/ijms17091388. 27563883 PMC5037668

[128] Sanchez Rodriguez MA, Zacarias Flores M, Arronte Rosales A, Mendoza Nunez VM. [Effect of hormone therapy with estrogens on oxidative stress and quality of life in postmenopausal women]. Ginecol Obstet Mex 81: 11–22, 2013. 23513399

[129] Ke RW, Todd Pace D, Ahokas RA. Effect of short-term hormone therapy on oxidative stress and endothelial function in African American and Caucasian postmenopausal women. Fertil Steril 79: 1118–1122, 2003. doi:10.1016/s0015-0282(03)00153-5. 12738505

[130] Tatsumi K, Pickett CK, Jacoby CR, Weil JV, Moore LG. Role of endogenous female hormones in hypoxic chemosensitivity. J Appl Physiol (1985) 83: 1706–1710, 1997. doi:10.1152/jappl.1997.83.5.1706. 9375342

[131] Marques DA, de Carvalho D, da Silva GSF, Szawka RE, Anselmo-Franci JA, Bicego KC, Gargaglioni LH. Influence of estrous cycle hormonal fluctuations and gonadal hormones on the ventilatory response to hypoxia in female rats. Pflugers Arch 469: 1277–1286, 2017. doi:10.1007/s00424-017-2022-y. 28660294

[132] Andersen ML, Bittencourt LR, Antunes IB, Tufik S. Effects of progesterone on sleep: a possible pharmacological treatment for sleep-breathing disorders? Curr Med Chem 13: 3575–3582, 2006. doi:10.2174/092986706779026200. 17168724

[133] Hunt BE, Taylor JA, Hamner JW, Gagnon M, Lipsitz LA. Estrogen replacement therapy improves baroreflex regulation of vascular sympathetic outflow in postmenopausal women. Circulation 103: 2909–2914, 2001. doi:10.1161/01.cir.103.24.2909. 11413079

[134] Vongpatanasin W, Tuncel M, Mansour Y, Arbique D, Victor RG. Transdermal estrogen replacement therapy decreases sympathetic activity in postmenopausal women. Circulation 103: 2903–2908, 2001. doi:10.1161/01.cir.103.24.2903. 11413078

[135] Xue B, Zhang Z, Beltz TG, Johnson RF, Guo F, Hay M, Johnson AK. Estrogen receptor-beta in the paraventricular nucleus and rostroventrolateral medulla plays an essential protective role in aldosterone/salt-induced hypertension in female rats. Hypertension 61: 1255–1262, 2013. doi:10.1161/HYPERTENSIONAHA.111.00903. 23608653 PMC3893074

[136] Nickenig G, Baumer AT, Grohe C, Kahlert S, Strehlow K, Rosenkranz S, Stablein A, Beckers F, Smits JF, Daemen MJ, Vetter H, Bohm M. Estrogen modulates AT1 receptor gene expression in vitro and in vivo. Circulation 97: 2197–2201, 1998. doi:10.1161/01.cir.97.22.2197. 9631868

[137] Xue B, Zhang Z, Beltz TG, Guo F, Hay M, Johnson AK. Estrogen regulation of the brain renin-angiotensin system in protection against angiotensin II-induced sensitization of hypertension. Am J Physiol Heart Circ Physiol 307: H191–H198, 2014. doi:10.1152/ajpheart.01012.2013. 24858844 PMC4101642

[138] Brosnihan KB, Li P, Ganten D, Ferrario CM. Estrogen protects transgenic hypertensive rats by shifting the vasoconstrictor-vasodilator balance of RAS. Am J Physiol Regul Integr Comp Physiol 273: R1908–R1915, 1997. doi:10.1152/ajpregu.1997.273.6.R1908. 9435644

[139] Iorga A, Cunningham CM, Moazeni S, Ruffenach G, Umar S, Eghbali M. The protective role of estrogen and estrogen receptors in cardiovascular disease and the controversial use of estrogen therapy. Biol Sex Differ 8: 33, 2017. doi:10.1186/s13293-017-0152-8. 29065927 PMC5655818

[140] Nevzati E, Shafighi M, Bakhtian KD, Treiber H, Fandino J, Fathi AR. Estrogen induces nitric oxide production via nitric oxide synthase activation in endothelial cells. Acta Neurochir Suppl 120: 141–145, 2015. doi:10.1007/978-3-319-04981-6_24. 25366614

[141] Barbagallo M, Dominguez LJ, Licata G, Shan J, Bing L, Karpinski E, Pang PK, Resnick LM. Vascular effects of progesterone: role of cellular calcium regulation. Hypertension 37: 142–147, 2001. doi:10.1161/01.hyp.37.1.142. 11208769

[142] Arnaud C, Bochaton T, Pépin JL, Belaidi E. Obstructive sleep apnoea and cardiovascular consequences: pathophysiological mechanisms. Arch Cardiovasc Dis 113: 350–358, 2020. doi:10.1016/j.acvd.2020.01.003. 32224049

[143] Delgado BJ, Lopez-Ojeda W. Estrogen. In: StatPearls (Internet). Treasure Island, FL: StatPearls Publishing, 2023.

[144] Lemke EA, Madsen LT, Dains JE. Vaginal testosterone for management of aromatase inhibitor-related sexual dysfunction: an integrative review. Oncol Nurs Forum 44: 296–301, 2017. doi:10.1188/17.ONF.296-301. 28635978

[145] Naessen T. The Heart and Estrogen/Progestin Replacement Study (HERS) in perspective–results not very surprising. Acta Obstet Gynecol Scand 79: 1037–1041, 2000. doi:10.1080/00016340009169258. 11130083

[146] Grady D, Brown JS, Vittinghoff E, Applegate W, Varner E, Snyder T; HERS Research Group. Postmenopausal hormones and incontinence: the Heart and Estrogen/Progestin Replacement Study. Obstet Gynecol 97: 116–120, 2001. 11152919 10.1016/s0029-7844(00)01115-7

[147] Aubrecht TG, Jenkins R, Magalang UJ, Nelson RJ. Influence of gonadal hormones on the behavioral effects of intermittent hypoxia in mice. Am J Physiol Regul Integr Comp Physiol 308: R489–R499, 2015. doi:10.1152/ajpregu.00379.2014. 25552660 PMC4360067

[148] Sneddon EA, Masters BM, Ream KD, Fennell KA, DeMedio JN, Cash MM, Hollingsworth BP, Pandrangi S, Thach CM, Shi H, Radke AK. Sex chromosome and gonadal hormone contributions to binge-like and aversion-resistant ethanol drinking behaviors in Four Core Genotypes mice. Front Psychiatry 14: 1098387, 2023. doi:10.3389/fpsyt.2023.1098387. 36960454 PMC10027717

[149] Sneddon EA, Rasizer LN, Cavalco NG, Jaymes AH, Ostlie NJ, Minshall BL, Masters BM, Hughes MR, Hrncir H, Arnold AP, Radke AK. Gonadal hormones and sex chromosome complement differentially contribute to ethanol intake, preference, and relapse-like behaviour in four core genotypes mice. Addict Biol 27: e13222, 2022. doi:10.1111/adb.13222. 36001422 PMC9413386

[150] Itoh Y, Mackie R, Kampf K, Domadia S, Brown JD, O'Neill R, Arnold AP. Four core genotypes mouse model: localization of the Sry transgene and bioassay for testicular hormone levels. BMC Res Notes 8: 69, 2015. doi:10.1186/s13104-015-0986-2. 25870930 PMC4354741

[151] Ghosh MK, Chen KE, Dill-Garlow R, Ma LJ, Yonezawa T, Itoh Y, Rivera L, Radecki KC, Wu QP, Arnold AP, Muller HK, Walker AM. Sex differences in the immune system become evident in the perinatal period in the four core genotypes mouse. Front Endocrinol (Lausanne) 12: 582614, 2021. doi:10.3389/fendo.2021.582614. 34122327 PMC8191418

[152] Corre C, Friedel M, Vousden DA, Metcalf A, Spring S, Qiu LR, Lerch JP, Palmert MR. Separate effects of sex hormones and sex chromosomes on brain structure and function revealed by high-resolution magnetic resonance imaging and spatial navigation assessment of the Four Core Genotype mouse model. Brain Struct Funct 221: 997–1016, 2016. doi:10.1007/s00429-014-0952-0. 25445841

[153] Arnold AP, Chen X, Grzybowski MN, Ryan JM, Sengelaub DR, Mohanroy T, Furlan VA, Grisham W, Malloy L, Takizawa A, Wiese CB, Vergnes L, Skaletsky H, Page DC, Reue K, Harley VR, Dwinell MR, Geurts AM. A “Four Core Genotypes” rat model to distinguish mechanisms underlying sex-biased phenotypes and diseases. *bioRxiv*, 2023. doi:10.1101/2023.02.09.527738.

